# A Surfactant Enables Efficient Membrane Spanning by Non-Aggregating DNA-Based Ion Channels

**DOI:** 10.3390/molecules27020578

**Published:** 2022-01-17

**Authors:** Diana Morzy, Michael Schaich, Ulrich F. Keyser

**Affiliations:** Cavendish Laboratory, University of Cambridge, JJ Thomson Avenue, Cambridge CB3 0HE, UK; diana.morzy@epfl.ch (D.M.); michael.schaich@cantab.net (M.S.)

**Keywords:** synthetic ion channels, nanopores, DNA nanotechnology, surfactant, insertion efficiency

## Abstract

DNA nanotechnology makes use of hydrophobically modified constructs to create synthetic membrane protein mimics. However, nucleic acid structures exhibit poor insertion efficiency, leading to a low activity of membrane-spanning DNA protein mimics. It is suggested that non-ionic surfactants improve insertion efficiency, partly by disrupting hydrophobicity-mediated clusters. Here, we employed confocal microscopy and single-molecule transmembrane current measurements to assess the effects of the non-ionic surfactant octylpolyoxyethylene (oPOE) on the clustering behavior and membrane activity of cholesterol-modified DNA nanostructures. Our findings uncover the role of aggregation in preventing bilayer interactions of hydrophobically decorated constructs, and we highlight that premixing DNA structures with the surfactant does not disrupt the cholesterol-mediated aggregates. However, we observed the surfactant’s strong insertion-facilitating effect, particularly when introduced to the sample separately from DNA. Critically, we report a highly efficient membrane-spanning DNA construct from combining a non-aggregating design with the addition of the oPOE surfactant.

## 1. Introduction

DNA nanotechnology has emerged as a promising tool for mimicking the functionality of proteins [1,2]. Synthetic membrane constructs have attracted particular attention, as natural ion channels [3], membrane enzymes [4], and bilayer-remodeling proteins [5] play a crucial role in every cell’s survival. By controlling DNA architecture at the nanoscale, DNA nanoengineering can offer novel gating mechanisms for synthetic ion channels [6,7], mimic enzymatic activity [8,9], and provide unique membrane-reshaping platforms [10,11]. However, with its strong charge rending it hydrophilic, DNA does not spontaneously interact with fluid lipid bilayers [12]. Strategies to overcome this problem have been developed, e.g., chemical engineering of a DNA backbone [13,14] or modification with a hydrophobic moiety [15,16,17,18,19]. In particular, DNA structures are often decorated with cholesterol molecules, which provide them with hydrophobicity driving a bilayer attachment [9,20,21,22]. Strategically modified DNA was shown to insert into bilayers, forming ion channels [7,9,21,23]. A library of membrane-spanning structures was developed: large DNA origami porins [24,25], smaller multi-helix bundles [7,11,23], and simple DNA duplexes [9,21,26].

Despite the hydrophobic modifications, DNA nanostructures suffer from low insertion efficiency [21]. One of the strategies employed to increase the insertion efficiency of DNA pores is premixing with a non-ionic surfactant such as octylpolyoxyethylene (oPOE) [7,8,21,27]. This is commonly used in membrane protein studies, where natural ion channels (e.g., OmpF) [28,29] are diluted with oPOE, which facilitates their incorporation into a model lipid bilayer. This strategy is based on the surfactant micelle formation around the protein, which mediates their bilayer insertion [30]. In research works featuring membrane insertion of DNA nanostructures, the surfactant was similarly premixed with the constructs, both in works featuring transmembrane current measurements [7,21,27] and a fluorescence-based assay [8]. However, it is not clear whether insertion is facilitated via the same mechanism used in the standard protocols employed in studying natural ion channels. It is rather suggested that the addition of oPOE impedes the formation of aggregates [31]. The clustering might indeed decrease DNA constructs’ membrane activity; therefore, the disruption of such aggregates has been the subject of numerous research reports [20,22,32,33]. Although DNA-surfactant complexes have been thoroughly studied for unmodified DNA and cationic detergents, particularly for gene delivery applications [34,35,36], the details of the interactions of cholesterol-modified DNA with non-ionic surfactants have not received much attention thus far.

Here, we studied the effects of oPOE surfactant on cholesterol-modified DNA nanostructures. We analyzed a library of constructs differing in the number of hydrophobic moieties and their structural design, resulting in various modes of aggregation. With the use of gel electrophoresis, confocal microscopy, and single-molecule transmembrane current measurements, we show that the addition of oPOE does not disrupt cholesterol-driven DNA clusters, but aids a bilayer insertion of the non-aggregating DNA architectures. In particular, we demonstrate that a separate addition of the surfactant to the sample improves the insertion efficiency more than when it is premixed with the DNA ion channel prior to incubating with the bilayer. We hypothesize that the insertion-facilitating effects of oPOE result mainly from its interactions with the lipid bilayer rather than DNA nanostructures. These results will help guide future protocols for measuring DNA membrane activity and deepen the understanding of DNA-lipid interactions. Additionally, we report that the non-aggregating DNA ion channel spans lipid bilayers with a high efficiency when aided by the addition of oPOE below its critical micelle concentration (cmc).

## 2. Results and Discussion

### 2.1. The Aggregation of Hydrophobically Modified DNA Nanostructures Is Determined by Their Design

**Figure 1:**
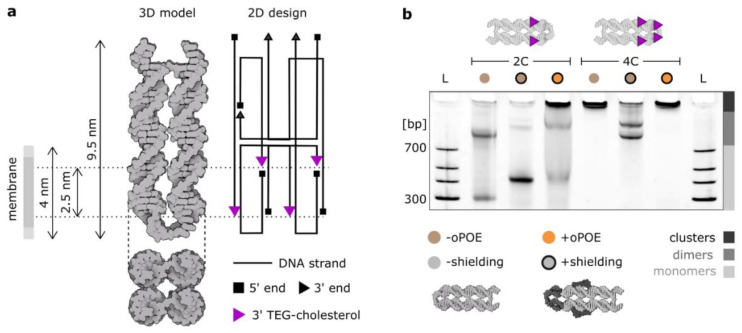
The aggregation of a DNA-based four-helix nanostructure is determined by its design. (**a**) The design and the atomic model of the four-helix membrane-spanning DNA construct. The 2D design was drawn using caDNAno software. Four potential cholesterol modification positions at the 3′ ends are labeled with purple triangles. The distance between the two modifiable planes matches the thickness of the hydrophobic core of a lipid bilayer. (**b**) Polyacrylamide gel electrophoresis (PAGE) of the two constructs used in this work: 4H labeled with either two (2C) or four cholesterol molecules (4C). Additional single-stranded overhangs were incorporated to prevent clustering of cholesterol molecules (“shielding”). The addition of 0.5% oPOE surfactant resulted in the majority of the con-structs being retained in the well, indicating a significant increase in size. For further analysis of the gel, see Figure S1.

In this work, we employed a cholesterol-modified four-helix (4H) DNA structure [22] (Figure 1a), whose geometry facilitates membrane spanning: the design inhibits hydrophobic modifications from orienting on the same side, making DNA insertion the only arrangement where all cholesterols can be embedded in the bilayer. The construct was designed to be as short as possible so as to avoid an excessive amount of charge to be positioned on a membrane. The 4H was designed to feature four modification positions in two planes, separated by 2.5–3 nm (≈thickness of a hydrophobic core of a membrane [37]) and shifted towards one end (to limit the amount of charged DNA that has to be pushed through the bilayer). Four 3′ ends that were used as modification positions for cholesterol “anchors” are highlighted in Figure 1a, which also gives an overview of the geometry of the construct.

Here, we discuss two major design parameters influencing the aggregating behavior of DNA ion channels: the number of hydrophobic anchors and the presence of single-stranded DNA overhangs in the proximity of cholesterol modifications, inhibiting their clustering: “shielding”. The effects of the former are intuitive: the more aggregation-driving molecules (here cholesterol), the stronger the aggregation. Note, however, that this effect is coupled with facilitating the membrane insertion, as more hydrophobic anchors will provide a stronger “pull” towards the bilayer. Thus, the number of hydrophobic modifications in particular should be well balanced. In this work, we compare the 4H structure with either two (2C) or four (4C) cholesterol modifications. The clustering effect is immediately visible when analyzing the PAGE presented in Figure 1b, where the electrophoretic mobility of unshielded 4C is reduced such that the structure is retained in the well. However, monomers of the unshielded 2C version migrate in the electric field, alongside a significant fraction of dimers.

“Shielding” is a previously described method of limiting the cholesterol-driven aggregation by introducing ssDNA overhangs, which, in an aqueous solution, can wrap around the hydrophobic modifications, preventing their strong clustering [22]. This method was shown to effectively decrease aggregation while preserving the membrane-anchoring properties of the design. Here, we introduced ssDNA loops at the terminal end of the structure (shielding two terminal cholesterols) and extended overhangs in the strands adjacent to the two cholesterols in the middle. The details of the sequences can be found in Table S1 and Figure S2. The results of our shielding strategy are prominent in the PAGE in Figure 1b, where both the 2C and 4C constructs change their clustering behavior, observed as the response to the electric field. The ratio between monomers and dimers in the 2C sample increased significantly when the shielding was introduced. Upon shielding, the monomer band migrated more slowly due to the increased size of the construct, yet it was much clearer and more pronounced, indicating that the majority of the structures were not aggregating. The signal of the 4C structure also changed, with most of structures migrating in the electric field. The sample features two bands, which we suggest correspond to the two possible dimers formed: either connected side-by-side or by the ends. Importantly, the clustering behavior of 4C structure was so strong that even the shielding strategy could not disrupt the formed dimers. This might be attributed to another important design-level parameter: the distribution of hydrophobic anchors [20]. Certain positions favor the formation of multimers, e.g., cholesterols at the structure’s end are very likely to induce dimerization. We observed such distribution-dependency of aggregation with 3C structures, labeled in various combinations of the four positions (Figure S3).

Additionally, the gel in Figure 1b features 4H premixed in a 1:1 ratio with 1% oPOE. After the addition of the surfactant, the concentration of DNA monomers in the solution decreased. The DNA bands containing the surfactant did not migrate in the electric field, which indicates the formation of large complexes, possibly DNA-trapping micelles, as oPOE was added above its critical micelle concentration (cmc oPOE = 0.15%) [38]. The gel in Figure 1b confirms that the oPOE interacts with cholesterol-modified DNA nanostructures. In the next part of the paper, we expand our studies on the observations from the gel in Figure 1b, investigating further the effects of cholesterol-induced aggregation, as well as analyzing the DNA nanostructures upon mixing with the surfactant.

### 2.2. The Aggregation of DNA Nanostructures Affects Their Membrane Interactions and Is Not Inhibited by the Addition of the Surfactant

We investigated the extent to which the cholesterol-mediated aggregation observed in solution impacts the membrane interactions of synthetic DNA ion channels. On one hand, embedding cholesterol into a lipid bilayer is a strongly favorable process, with a free energy of insertion ≈ −75 kJ/mol [21,24]. We considered whether cholesterol clusters disassemble in order to enable membrane insertion. Indeed, it has been shown previously that cholesterol-mediated DNA aggregates can be broken in the presence of lipid bilayers, allowing monomer interactions with the membrane [20,39]. However, the same report also showed that this effect is hindered when the number of cholesterol modifications increases, resulting in a non-uniform membrane coating.

**Figure 2:**
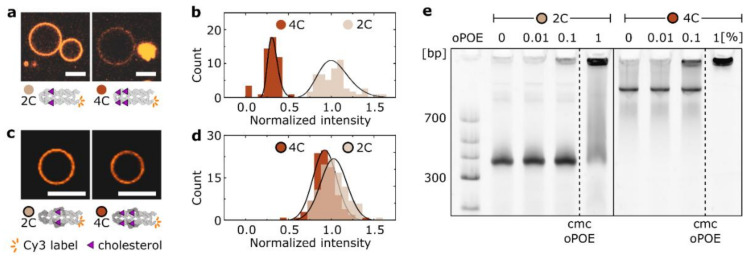
Membrane interactions of cholesterol-modified DNA structures depend on their clustering, which is not impaired in the presence of a surfactant. (**a**) Representative confocal micrographs of POPC vesicles coated with Cy3-labeled, non-shielded 2C and 4C constructs. Strong clustering behavior of 4C manifests in the presence of large aggregates attached to the membrane. Scale bars: 10 µm. (**b**) Histograms of the fluorescence intensity of the vesicle coating represented in panel (**a**). The data are normalized to the peak of the normal distribution fitted to the values collected for the 2C sample. The number of measured vesicles: N_2C_ = 58, N_4C_ = 54. (**c**,**d**) Microscopy analysis analogous to the data presented in panels (**a**,**b**), collected for shielded DNA constructs. Scale bars: 10 µm. The number of measured vesicles: N_2C_ = 103, N_4C_ = 96. (**e**) PAGE of shielded 2C and 4C in the presence of an increasing concentration of oPOE in the sample: 0, 0.01, 0.1, and 1%. Dashed line represents cmc of oPOE = 0.15% [38]. For a detailed analysis of the gel, see Figure S4.

We investigated this further by imaging Cy3-labeled 4H constructs in the presence of giant unilamellar vesicles (GUVs) made with POPC lipids. We observed that a fluorescent DNA coating for non-shielded structures indicates their interactions with bilayers. Even though the 2C constructs carried less hydrophobicity, they formed a much more prominent coating, while the sample of 4C had a weaker membrane signal and featured visible aggregates (Figure 2a). The quantitative analysis of the fluorescence intensity of the DNA coatings (Figure 2b) shows the difference in the degree of attachment, which we deduce as resulting from 4C forming much larger and more stable cholesterol-induced clusters. This discrepancy in the coating observed for non-shielded structures was also noticeable for the shielded ones (Figure 2c,d), but it was far less prominent due to the clustering being hindered by the overhanging loops. The results presented in Figure 2a–d indicate that even though cholesterol provides the “driving force” for the membrane interactions, increasing its amount does not always lead to stronger bilayer attachment. We hypothesize that this finding will be reflected in the membrane activity of our functional ion channels.

One of the methods used when studying bilayer insertion of DNA nanoengineered structures is their premixing with a surfactant [8,21,31]. We prepared mixtures of shielded 4H structures and the oPOE surfactant and observed their electrophoretic mobility with PAGE (Figure 2e). The surfactant additions of 0, 0.01, 0.1, and 1% were applied to 2C and 4C constructs. For both of the DNA ion channels, we observed no disruption of clusters upon mixing with the oPOE. On the contrary, at 1% oPOE, we observed the formation of large structures immobile on the gel. As the cmc of oPOE was 0.15%, the band retained in the well can be attributed to micelles capturing the DNA. However, even at concentrations below cmc, the addition of the surfactant resulted in an increased number of aggregates, as analyzed in more detail in Figure S4. We observed the same trend when premixing 4C with another surfactant: negatively charged SDS (Figure S5), which has been previously employed to limit the intermolecular interactions of cholesterol-labeled DNA origami structures [27]. Additionally, we obtained consistent results with dynamic light scattering (DLS) measurements (Figure S6), where, for both of the constructs, the count of structures larger than monomers was higher after the addition of the surfactant below its cmc. Thus, we suggest that the presence of a surfactant may facilitate cholesterol-mediated clustering but does not prevent it. A lack of any aggregation-inhibiting effect of the added oPOE on formed DNA constructs has been observed previously [31], although the report shows that cholesterol-induced clusters were not observed when oPOE was introduced during folding of DNA nanostructures.

### 2.3. The Addition of Surfactant below Its Cmc Improved the Insertion Efficiency of Non-Aggregating DNA Constructs

**Figure 3:**
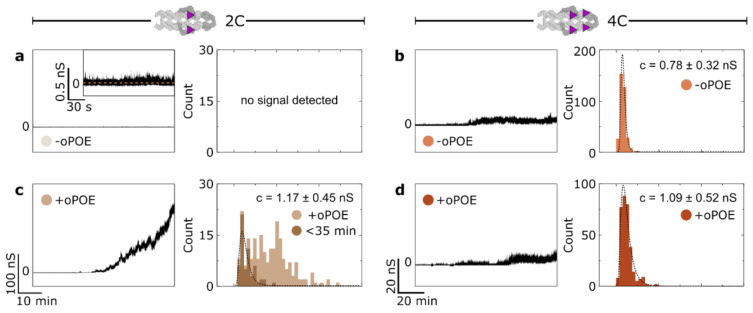
Ion channel formation by DNA constructs is highly efficient when non-clustering structures are aided by the addition of the surfactant. The conductance across a DPhPC bilayer was measured in 0.5 M KCl in the presence of (**a**,**c**) 2C and (**b**,**d**) 4C structures, with either an addition of 0.01% oPOE (+oPOE) or with no surfactant added (−oPOE). (**a**,**b**) Representative ionic conductance traces measured for the 2C (**a**) and 4C (**b**) structures in the absence of oPOE, alongside histograms of reported signals collected from all measurements. The inset in (**a**) shows a perturbated conductance signal with no clearly defined stepwise increase in the measured current. (**c**,**d**) Data analogous to panels (**a**,**b**), obtained after the addition of 0.01% oPOE. The histogram for the 2C sample (**c**) highlights the signal obtained throughout first 35 min of each run when the identification of single-molecule-induced conductance steps was possible. The dashed lines represent lognormal fits with the conductance peak values stated on each plot. The error values represent the standard deviation. The data were collected from three independent experiments, each at least one hour-long. Non-modified, non-inserting control structures produced no changes in current flow, similar to the sole addition of the surfactant onto the membrane (Figure S7). Additional examples of traces recorded for all the samples are shown in Figure S8. The number of detected events: N_2C(−oPOE)_ = 0, N_2C(+oPOE)_ = 212, N_2C(<35min)_ = 58, N_4C(−oPOE)_ = 344, N_4C(+oPOE)_ = 331.

We employed single-molecule transmembrane current measurements to monitor 4H structures’ membrane insertion. The experiments were performed on a DPhPC membrane in 0.5 M KCl and 25 mM HEPES (pH 7.0), under the voltage of 50 mV, with a final DNA concentration of 10 nM. Where stated, an addition of oPOE to a final concentration of 0.01% was made. The current traces collected in the presence of the unmodified (0C) control structure, as well as after the addition of only oPOE, produced no conductance changes (Figure S7), as was reported previously [21]. Initially, we observed DNA-induced changes in the membrane conductance in the absence of the surfactant. While the 2C construct produced a transient, weak signal only, the 4C channel caused significant perturbation in the bilayer’s ion permeability (Figure 3a–b). This observation indicates that an increased number of hydrophobic anchors results in higher membrane activity, as was expected in light of the hydrophobicity-driven lipid interactions of the designed constructs. However, we note that in neither case was the insertion efficiency high. Even the 4C structures caused a signal that oscillated not far above the baseline, indicating that few constructs were spanning the membrane at any given time.

The samples in which the oPOE surfactant was added into the chamber before starting the measurement (at a final concentration of 0.01%) showed an entirely different trend. The surfactant’s effect on the 4C sample behavior was negligible; however, its addition to the 2C samples resulted in a significant change in the conductance of the bilayer (Figure 3c,d). Despite 2C carrying less insertion-driving cholesterol molecules, in the presence of the surfactant, it spanned the membrane much more efficiently than the 4C construct. Thus, we confirm that the addition of oPOE below its cmc results in higher insertion efficiency, at least for the non-aggregating structures. Additional examples of the obtained traces are shown in Figure S8. A more detailed analysis of the traces obtained for the highly efficient 2C sample are presented in Figure S9 and Figure S10, featuring an example of well-defined, single-molecule steps, as well as I-V curve analysis, respectively.

To gain further insight into the insertion process, we analyzed the current traces obtained for membrane-spanning constructs using the “single channel search” feature of the pCLAMP software suite to study the magnitude of discreet conductance changes throughout the experiment. Figure 3 presents histograms of the obtained signals alongside the respective conductance traces. The highly efficient spanning of 2C consisted of a wide distribution of conductance steps (Figure 3c). Plotting the conductance changes vs. time revealed that the observed signals were steadily increasing (Figure S11). We suggest that, with time, a growing number of constructs insert simultaneously into the membrane. With multiple coinciding insertions, the time resolution of our experiment prevented us from clearly distinguishing single-channel events. However, in Figure 3c, we have highlighted the values collected for the initial 35 min of each run, with the assumption that, until this time, mostly single-molecule events are observed. The resulting histogram was fitted with a lognormal function, peaking at 1.17 ± 0.45 nS, which we attribute to the mean conductance of a single pore-forming 2C construct in the presence of oPOE.

On the other hand, the signals collected for 4C yielded narrow histograms for both samples without (Figure 3b) and with oPOE (Figure 3d). The peak conductance for the structure observed in the presence of the surfactant was 1.09 ± 0.52 nS, a value closely matching that obtained for the 2C structures in the same conditions. This was to be expected, as the dimensions of both of the constructs were the same. The slightly lower (≈7%) conductance of 4C might be attributed to the higher hydrophobicity of the DNA–lipids interface caused by additional cholesterol molecules impeding this pathway of the ion flow [9]. On the other hand, 4C measured in the absence of oPOE produced a histogram with a peak at 0.78 ± 0.32 nS, which is substantially (≈31%) lower than for both samples with the surfactant. We hypothesize that surfactant molecules surround the pore-forming DNA, probably as a rim of a toroidal pore [40]. Their arrangement at the DNA-lipid interface resulted in an increased DNA-induced conductance, which in turn indicates that they play a role in pore formation.

Here, we speculate that the aggregation of DNA structures is one of the dominant aspects influencing their insertion efficiency. While 2C exists in a solution in a monomeric state, 4C aggregates spontaneously into large clusters. These clusters do not disassemble in the presence of a bilayer, and thus the concentration of membrane-active monomeric structures is effectively lower. Even though oPOE addition into the chamber aids membrane insertion, its effects were not observed for these strongly clustered 4C constructs. This agrees with our observation of the lack of oPOE’s aggregation-inhibiting effects. Furthermore, we have strong indications that its premixing with cholesterol-modified DNA structures might facilitate aggregation, as seen from the current measurements for 2C mixed with oPOE before its addition to the chamber (Figure S12). Although this sample showed much improvement in insertion efficiency as compared with the one with no oPOE, the increase in the conductance did not rise to as high values as in the case of a separate addition (Figure 3c), but rapidly plateaued instead.

To better understand the role of oPOE in our system, we performed fluorescence recovery after photobleaching (FRAP) measurements [41] to obtain diffusion coefficients of lipids and bilayer-attached DNA with and without the addition of the surfactant (Figure S13). Even though DNA mobility did not seem to be greatly affected by the surfactant, the lipids show a decrease in their diffusivity in the presence of oPOE. This effect on lipid mobility, as well as the less prominent insertion improvement for the premixed surfactant (Figure S12) and the observed aggregation in the premixed samples (Figure 2e), suggest that the mechanism through which oPOE facilitates membrane insertion of DNA nanostructures is by interacting with the bilayer rather than with constructs. This is further supported by observing the membrane attachment of the Cy3-labeled constructs with and without 0.01% oPOE (Figure S13). No clear differences were observed, suggesting that oPOE at this concentration does not have a large influence over the distribution of DNA structures on the bilayer, but rather interacts with the system on another scale. We hypothesize that oPOE inserts into a bilayer and facilitates the formation of a toroidal pore: since strong curvatures release the strain that surfactant insertion causes, its presence can result in favorable membrane-spanning of DNA nanostructures. As polyoxyethylenes were shown to induce membrane thinning [42], and through this facilitate the electroporation of cell membranes [43], we hypothesize that such disruption might be the main mechanism of oPOE activity in the DNA-lipid system.

## 3. Materials and Methods

### 3.1. DNA Nanostructure Assembly

All the reagents used in this work were acquired from Sigma Aldrich (St. Louis, Mo, USA), unless stated otherwise. Each single strand was analyzed using the NUPACK suite [44] in order to prevent the formation of secondary structures and to ensure a sufficient yield of folding. The sequences are shown in Table S1. All the oligonucleotides were provided by Integrated DNA Technologies, Inc (Coralville, Lowa, USA), including those with the 3′ end modified with either Cy3 fluorophore or TEG-cholesterol (both HPLC purified). All the strands were dissolved to a final concentration of 100 μM: unmodified ones in IDTE buffer (10 mM Tris, 0.1 mM EDTA (Ethylenediaminetetraacetic acid), pH 8.0) and the modified ones in Milli-Q purified water. The strands were then stored at 4 °C, except for the dye-modified ones, which were stored as aliquots at −20 °C. In order to fold the designed structures, the strands were mixed to a final concentration of 1 μM in TE20 buffer (10 mM Tris, 1 mM EDTA, 20 mM Mg^2+^, pH 8.0), with cholesterol-modified strands heated beforehand at 70 °C for 10 min. The folding protocol was initiated by preheating the oligonucleotide mixture to 85 °C, ensuring complete strand separation, followed by cooling to 25 °C for 18 h using an Applied Biosystems™ ProFlex™ PCR thermal cycler (Thermo Fisher Scientific, Inc., Waltham, MA, USA). The folded structures were all stored at 4 °C.

### 3.2. Ionic Current Measurements

Ionic current measurements were carried out as described previously [21], using solvent-containing membranes. Hexadecane (1% in pentane) was used to coat both sides of a hole (Ø = 0.15 mm) in the foil dividing the cis and trans chambers of the Teflon cuvette. After 5 min of incubation, 700 μL of 0.5 M KCl and 25 mM HEPES (4-(2-hydroxyethyl)-1-piperazineethanesulfonic acid) (pH 7.0) were added to each chamber. A total of 5 μL of 5 mg/mL DPhPC lipids (1,2-diphytanoyl-sn-glycero-3-phosphocholine, Avanti Polar Lipids) in pentane were added dropwise to each side, then the whole solution was gently pipetted up and down until the membrane was formed. The current data were acquired at a sampling rate of 1 kHz using an Axon^TM^ Axopatch 200B amplifier. After membrane formation, the DNA structures were added to the cis side at a final concentration of 10nM. Where stated, oPOE was also added, separately, to a final concentration of 0.01%. The ionic current under 50 mV voltage across the membrane was recorded. The experiments were repeated three times for each construct and run for at least an hour each. Clampex and Clampfit software was used to gather and analyze the data: the “single channel search” tool of Clampfit was used to automatically detect events. Each dataset was analyzed using the same settings: ignoring effects of <10 ms and only detecting single-level changes, with the level initialized at 25 pA (0.5 nS). Assuming an ohmic behavior of the formed pores, conductance (*c*) was reported as the recorded current (*I*) by voltage (*V*), according to Equation (1).

(1) $$c=\frac{I}{V}\;$$

Lognormal distribution curves were fitted to the obtained histograms, following Function (2).

(2) $$y=y_{0}+\frac{A}{\sqrt{2\pi }wx}e^{\frac{-{\left[ln\frac{x}{x_{c}}\right]}^{2}}{2w^{2}}}\;$$

where $y_{0}$—offset,$\;x_{c}$—center, w—log standard deviation, A—area.

The standard deviation was calculated using Formula (3). Both formulae, reported by Origin software, were used for plotting the data.

(3) $$∆y=e^{ln\left(x_{c}\right)+0.5w^{2}}\sqrt{e^{w^{2}}-1}$$

### 3.3. Native Polyacrylamide Gel Electrophoresis (PAGE)

The polyacrylamide gels were prepared at a concentration of 10% polyacrylamide, 0.5x TBE (Tris, borate, EDTA), and 11 mM MgCl_2_. An addition of 0.01 vol% ammonium persulfate (APS) (10%) and 6.7 × 10^−4^% N,N,N’,N’Tetramethylethylenediamine (TEMED) was used to initialize polymerization, which proceeded for half an hour. A total of 2 μL of a DNA sample was mixed with 0.4 μL of 6× loading dye (15% Ficoll R400 and 0.9% Orange G diluted in Mili-Q water), and then 2 μL of the sample was loaded into the well. A Thermo Scientific^TM^ GeneRuler Low Range ladder (Thermo Fisher Scientific, Inc., Waltham, MA, USA) was used as a reference. The gel was run in a Mini-PROTEAN R Tetra Cell (Bio-Rad), in 0.5× TBE with 11 mM MgCl_2_ at 100 mV for 90 min. After this time, the gel was immersed for 10 min in GelRed (Biotium) in order to stain the DNA. The imaging was performed on a UVP GelDoc-It TM (Fisher Scientific, Hampton, US), and FIJI was used to process the images [45,46].

### 3.4. Confocal Microscopy Imaging

Giant unilamellar vesicles were prepared with electroformation, as reported previously [8,9,12]. POPC (1-palmitoyl-2-oleoyl-glycero-3-phosphocholine) and NBD-PC lipids (1-palmitoyl-2-{6-[(7-nitro-2-1,3-benzoxadiazol-4-yl)amino]hexanoyl}-sn-glycero-3-phosphocholine), both acquired from Avanti^®^ Polar Lipids, Inc. (Alabaster, AL, USA) were used in a ratio of 200:1, with a final concentration of 5 mg/mL in chloroform. A total of 600 μL of 1 M sorbitol in 200 mM sucrose was used as a buffer. The osmolality of the buffer was around 1200 mOsm, with all the dilution buffers used in the experiments adjusted accordingly. All the buffers were adjusted to pH 7.5 (using sodium hydroxide and hydrochloride solutions). Confocal microscopy images were acquired on an Olympus (Shinjuku City, Japan) FluoView filter-based FV1200F-IX83 laser scanning microscope using a 60× oil immersion objective (UPLSAPO60XO/1.35). Cy3 excitation was performed using a 1.5 mW 543 nm HeNe laser at 1% laser power, with the emission collected between 560 and 590 nm. FIJI was used to analyze the images.

FRAP measurements were performed following the protocol described previously [41]. Briefly, the field of view was focused on the bottom of a GUV. Using the FRAP function of the microscope’s software (“tornado mode”), a spot of Ø = 4 μm was bleached for 0.1 s with 98% laser power. The fluorescence recovery was recorded for 100 frames (2 μs/pixel exposure). An exponential function of Formula (4) was fit to the fractional recovery curve of each vesicle. The half-life recovery time *t*_1/2_ was extracted to calculate the lipid lateral diffusion coefficient according to Formula (5).

(4) $$y=y_{0}\left(1-\exp\left(-at\right)\right)$$

(5) $$D=0.224\frac{w^{2}}{t_{\frac{1}{2}}}$$

## 4. Conclusions

Below its cmc, surfactant addition resulted in a significant increase in the insertion efficiency of 2C, which, in the solution, existed in a monomeric state. While investigating the effects of the surfactant addition on DNA insertion efficiency, we confirmed the importance of aggregation in determining the outcome of the membrane insertion process. The clustering of hydrophobically modified DNA constructs has already been recognized as an important parameter determining DNA membrane activity and, as such, has been the subject of numerous studies [20,22,32,33]. Importantly, it was reported that aggregation lowers the membrane insertion rate of DNA structures [20,47], and our observations showed similar effects even for shielded structures and in the presence of oPOE surfactants. Thus, various methods have been employed to minimize the impact of clustering [33]: optimizing the separation between cholesterols and decreasing the length of a spacer, folding in the presence of the surfactant [27,31,33], adding cholesterol in the second folding step [24,25,33], and, finally, the shielding technique used in this work [22,33,48,49].

Previously published studies [20,50], as well as our results, suggest that cholesterol-mediated clusters can spontaneously disassemble in the presence of a lipid bilayer. This phenomenon is thought to be driven by the high affinity of cholesterol towards a lipid environment. Future work based on imaging techniques and atomic force microscopy should allow the visualization of phenomena occurring on the lipid surfaces, particularly for DNA-based ion channels differing in cholesterol number and position and in the presence or absence of surfactants such as oPOE. Such studies require a simultaneous observation of changes in the membrane activity, as the lipid interactions of DNA nanoengineered constructs are driven by, and are thus strongly dependent on, their hydrophobic modifications.

Our results illustrate this dependency and its importance in designing synthetic ion channels: in the absence of the surfactant, the construct with more hydrophobic modifications (4C) was more successful in membrane spanning than the one with less cholesterols (2C), since cholesterol provides a driving force for the insertion. However, neither could be called an efficiently inserting structure. On the other hand, when aided by oPOE, the non-aggregating 2C was far more efficient in increasing membrane’s conductance in comparison to that observed for 4C. The effects of the surfactant on the insertion of the latter were minimal, which agrees with our observations that surfactant addition does not disrupt the aggregates. The results suggest that oPOE disorders the membrane, facilitating the insertion of pre-existing DNA monomers. One of the remaining challenges is to produce a detailed molecular description of the interactions between a non-ionic surfactant and cholesterol-modified DNA. In particular, whether premixing above the cmc results in the formation of DNA-containing micelles and if such micelles can mediate bilayer insertion remain unanswered. Additi0onally, a molecular-level understanding of the non-ionic surfactant’s influence on the aggregating behavior will offer further insight.

DNA nanotechnology offers a toolbox for the construction of membrane structures with customized geometry and a variety of gating mechanisms. Overcoming their low insertion efficiency will make their use more versatile. Here, we demonstrated that the addition of the surfactant can facilitate DNA constructs’ membrane spanning. Although not suitable for in vivo applications, this will provide new perspectives for nanopore use, including DNA and protein sequencing [27,49,51,52], and will enable the modeling and fine-tuning of the designs of DNA-based components of synthetic cells, such as membrane enzymes or ion channels [1,53,54]. Our findings shed light on the DNA membrane insertion issue, particularly by showing the importance of aggregation in membrane activity and helping to more deeply understand the action of surfactants in the DNA-lipid system. Importantly, we report the high-efficiency membrane insertion of the non-aggregating 2C DNA construct after the addition of oPOE below its cmc. This structure can be introduced as a spontaneously inserting ion channel, but is also a blueprint, guiding future designs of DNA membrane nanopores tailored to a variety of specific tasks.

## Data Availability

All of the raw data files are available on request.

## Supplementary Materials

All of the referenced supporting information can be downloaded, Figure S1: Intensity profiles of the wells of PAGE presented in Figure 1b, Figure S1: A 2D design (caDNAno) of 4H with labeled strands (sequences in Table S1), Figure S2: PAGE analysis of additional 4H structures: unlabeled 0C (control) and three-cholesterol 3C, alongside 2C (with and without Cy5 label) and 4C., Figure S3: Intensity profiles of the wells of the PAGE experiment presented in Figure 2d, Figure S4: PAGE analysis of shielded 4C 4H construct mixed at different concentrations of SDS: below and above its cmc (0.23%). The gel (a) and corresponding intensity profiles (b), Figure S5: Dynamic light scattering (DLS) size measurements of 4H: 2C and 4C structures in 0, 0.01, and 0.1% oPOE. Peaks collected for 0C (no oPOE) and 0.1% oPOE (no DNA) are shown for comparison, Figure S6: Representative conductance traces of the control samples with no cholesterol modifications (0C) or membrane after the addition of oPOE, Figure S7: Additional examples of conductance traces collected for the 4H structures with (+oPOE) and without (−oPOE) the addition of surfactant to the chamber, Figure S8: An example of two consecutive, well-defined insertions (a), followed by the nanostructures exiting the bilayer, illustrating the transient behavior of the pores. The further part of the traces is an example of the “gating” behavior (b) observed earlier for similar structures, Figure S9: I-V curves collected for a shielded 2C structure during the measurement, Figure S10: Scatter plots of discrete conductance changes (attributed to membrane insertions) vs. time from the beginning of the measurement, collected for shielded (a) 2C and (b) 4C samples, Figure S11: Conductance traces for 4H structures premixed with oPOE before the experiment, Figure S12: Confocal microscopy analysis of oPOE effects on a DNA–lipid system. (a) Box plots of diffusion coefficients collected for NBD-labelled PC lipids (no DNA addition) and Cy3-labelled DNA coating of the POPC vesicles, using fluorescence recovery after the photobleaching (FRAP) method, measured at room temperature (b) Representative micrographs of Cy3-labelled structures coating POPC vesicles in the absence and presence of 0.01% oPOE, Table S1: Sequences of DNA strands forming 4H structures.

## Sample Availability

Samples of the 4H DNA structures are available from the authors.
