# Supplementary material for: A Surfactant Enables Efficient Membrane Spanning by Non-Aggregating DNA-Based Ion Channels
Source: Molecules. 2022 Jan 17;27(2):578. doi: 10.3390/molecules27020578 (PMC8779190; doi:10.3390/molecules27020578)
Supplement: Supplementary file 1 [file molecules-27-00578-s001.zip › molecules-1542408-supplementary.pdf]

# Supplementary Information for

# A Surfactant Enables Efficient Membrane Spanning by Non-aggregating DNA-based Ion Channels

**Diana Morzy[1,†], Michael Schaich[1] and Ulrich F. Keyser[1,*]**

[1] Cavendish Laboratory, University of Cambridge, JJ Thomson Avenue, CB3 0HE Cambridge, United Kingdom; diana.morzy@epfl.ch (D.M.); michaelschaich@hotmail.com (M.S.)

* Correspondence: ufk20@cam.ac.uk

† Current address: Programmable Biomaterials Laboratory, School of Engineering, École Polytechnique Fédérale de Lausanne, Route Cantonale, 1015 Lausanne, Switzerland

## Contents

Figure S1 ........ **Error! Bookmark not defined.**

Figure S2 ........ **Error! Bookmark not defined.**

Figure S3 ........ **Error! Bookmark not defined.**

Figure S4 ........ **Error! Bookmark not defined.**

Figure S5 ........ **Error! Bookmark not defined.**

Figure S6 ........ **Error! Bookmark not defined.**

Figure S7 ........ **Error! Bookmark not defined.**

Figure S8 ........ **Error! Bookmark not defined.**

Figure S9 ........ **Error! Bookmark not defined.**

Figure S10 ........ **Error! Bookmark not defined.**

Figure S11 ........ **Error! Bookmark not defined.**

Figure S12 ........ **Error! Bookmark not defined.**

Figure S13 ........ **Error! Bookmark not defined.**

Table S1 ........ 15

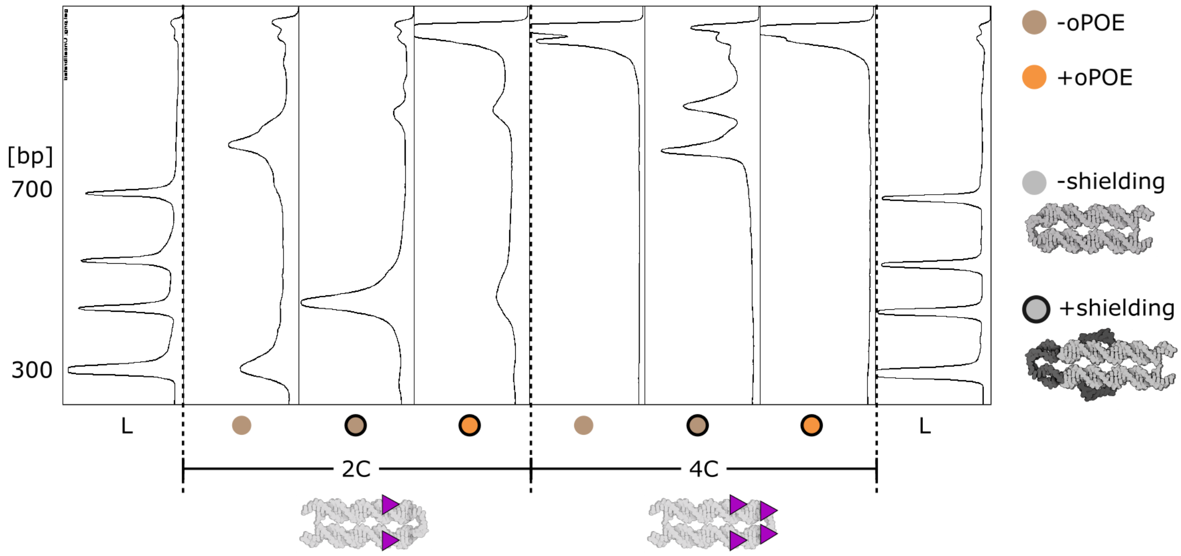


**Figure S1.** Intensity profiles of the wells of PAGE presented in Fig. 1b.

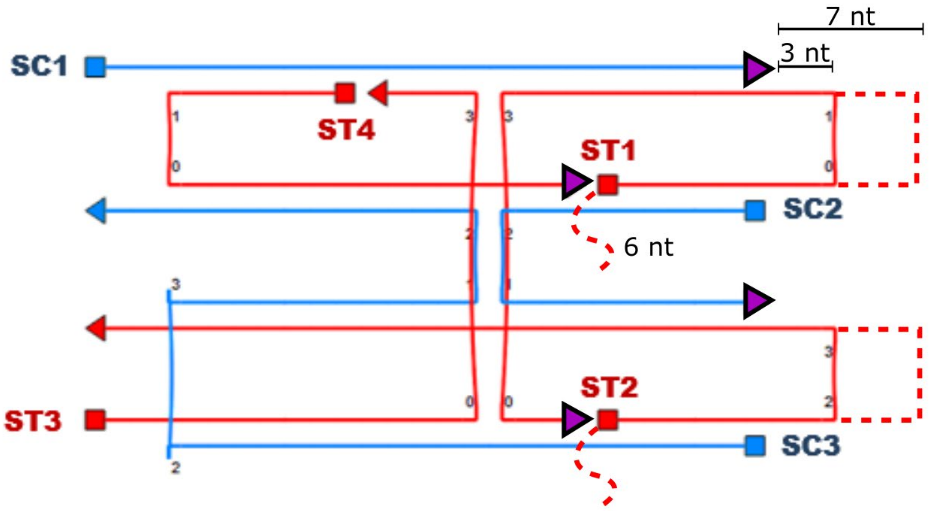


**Figure S2.** A 2D design (caDNAno) of 4H with labeled strands (sequences in Table S1). The dashed lines represent the extensions that formed "shielding" of cholesterol modifications, represented as purple triangles (3' end modifications).

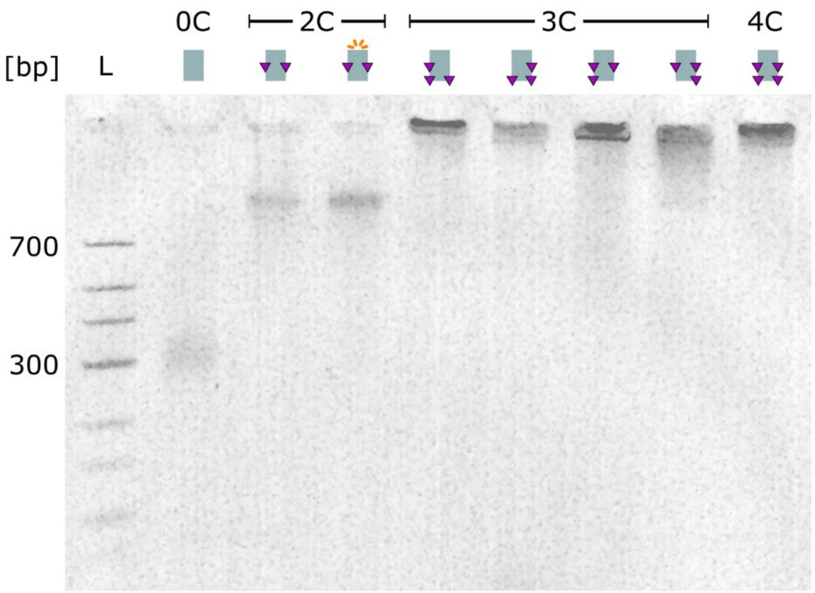


**Figure S3.** PAGE analysis of additional 4H structures: unlabeled 0C (control) and three-cholesterol 3C, alongside 2C (with and without Cy5 label) and 4C. All the constructs presented here are without shielding. The 3C structures were labelled in various configurations, and we note differences in their clustering, suggesting that the position of the hydrophobic modifications plays an important role in nanoengineered, membrane-spanning DNA structures.

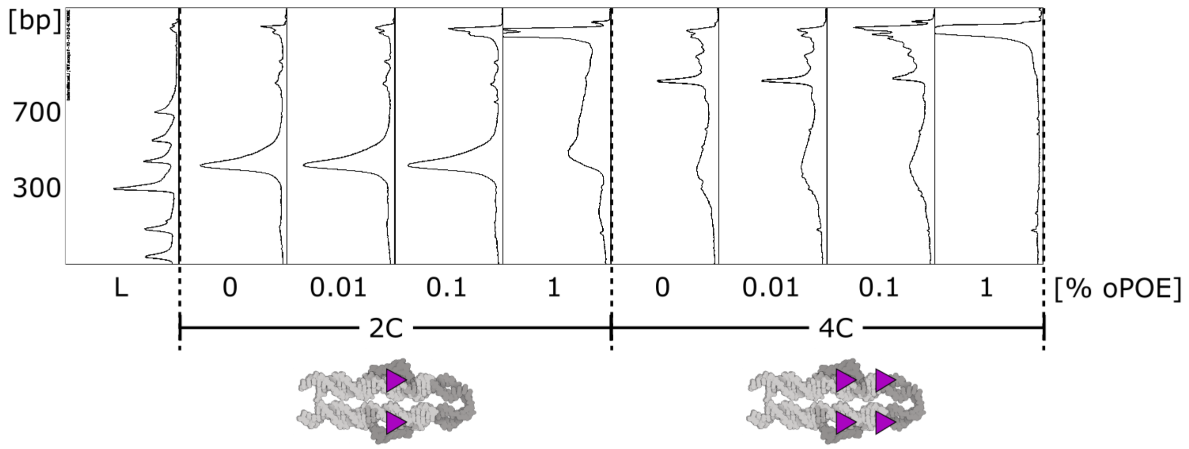


**Figure S4.** Intensity profiles of the wells of the PAGE experiment presented in Fig. 2d. Even below its cmc (0.15%), the addition of oPOE resulted in an increased intensity in the bands attributed to clusters (particularly at 0.1%).

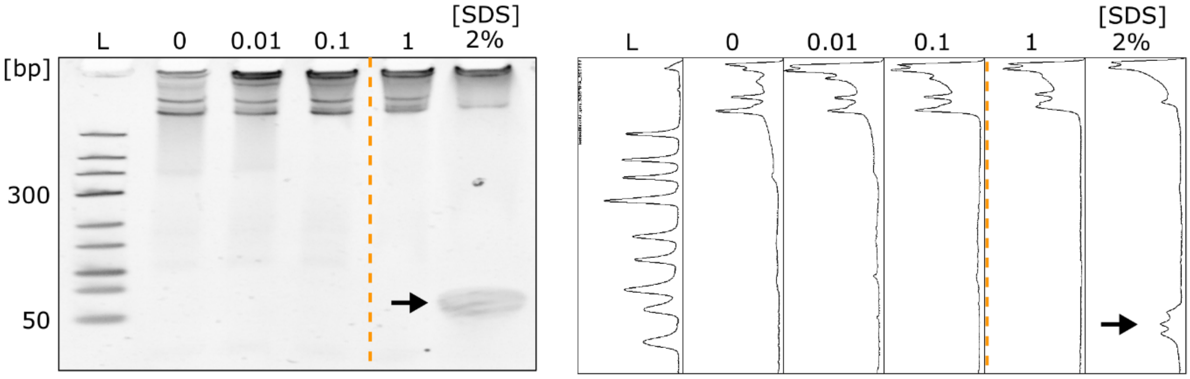


**Figure S5.** PAGE analysis of shielded 4C 4H construct mixed at different concentrations of SDS: below and above its cmc (0.23%)[9]. The gel (a) and corresponding intensity profiles (b). The dashed line separates lanes with [SDS] < cmc (left) and [SDS] > cmc (right). An additional band appearing at [SDS] = 2%, presumably containing SDS micelles, is marked with a black arrow.

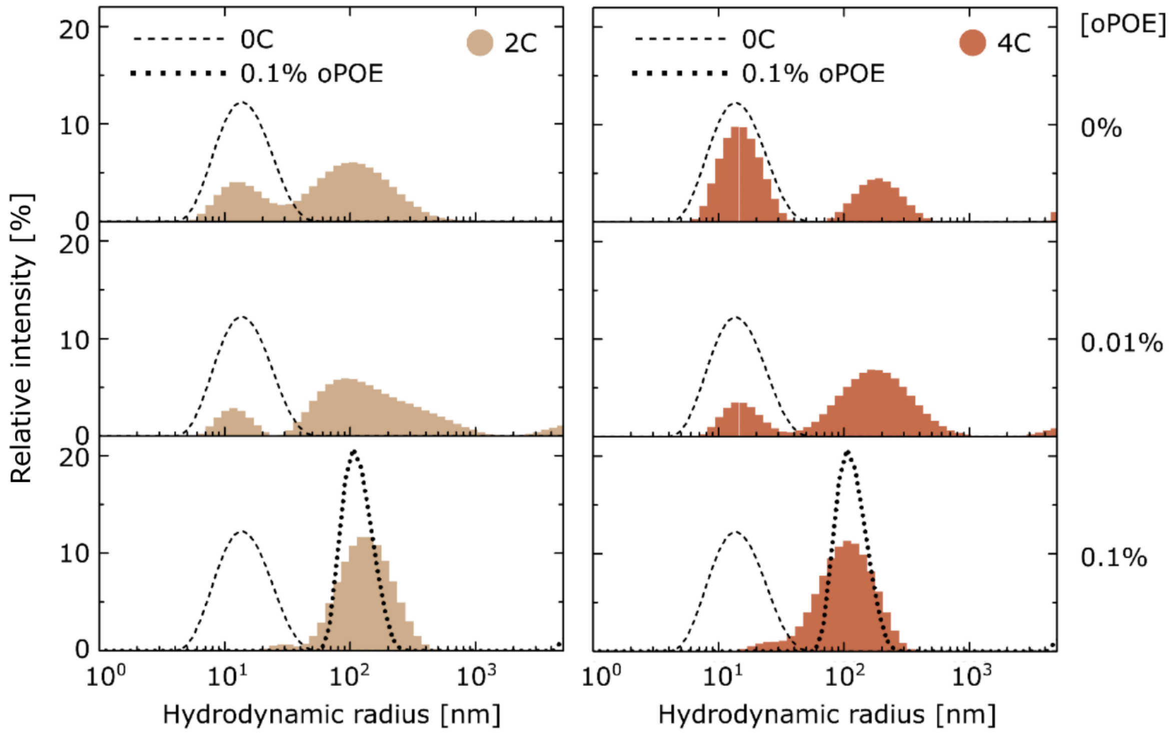


**Figure S6.** Dynamic light scattering (DLS) size measurements of 4H: 2C and 4C structures in 0, 0.01, and 0.1% oPOE. Peaks collected for 0C (no oPOE) and 0.1% oPOE (no DNA) are shown for comparison. The peak representing the monomers (as compared with non-aggregating 0C) decreased upon addition of oPOE below its cmc, and can no longer be observed at 0.1% surfactant.

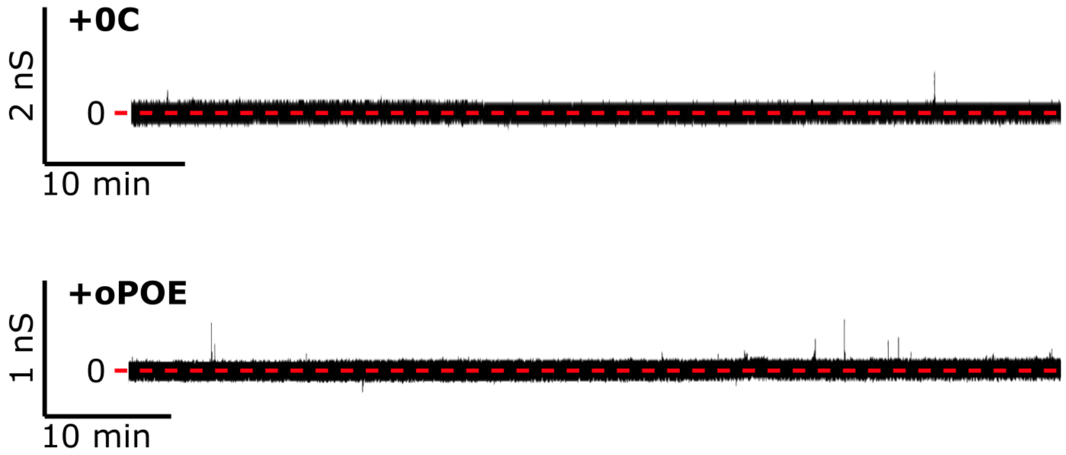


**Figure S7.** Representative conductance traces of the control samples with no cholesterol modifications (0C) or membrane after the addition of oPOE. These controls were repeated three times; each run lasted at least an hour. No action of unmodified DNA or oPOE was observed previously [2,5].

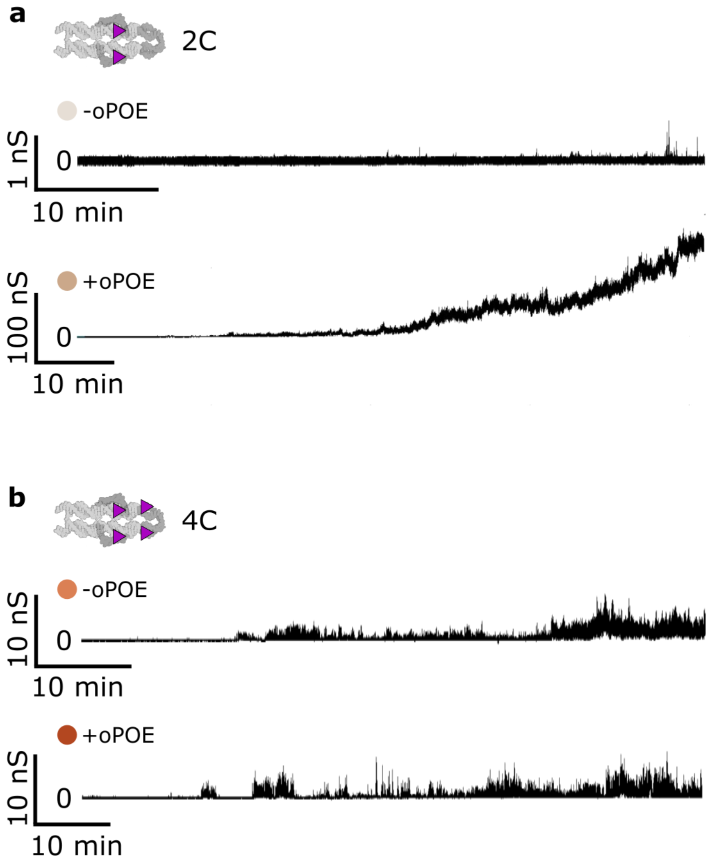


**Figure S8.** Additional examples of conductance traces collected for the 4H structures with (+oPOE) and without (-oPOE) the addition of surfactant to the chamber.

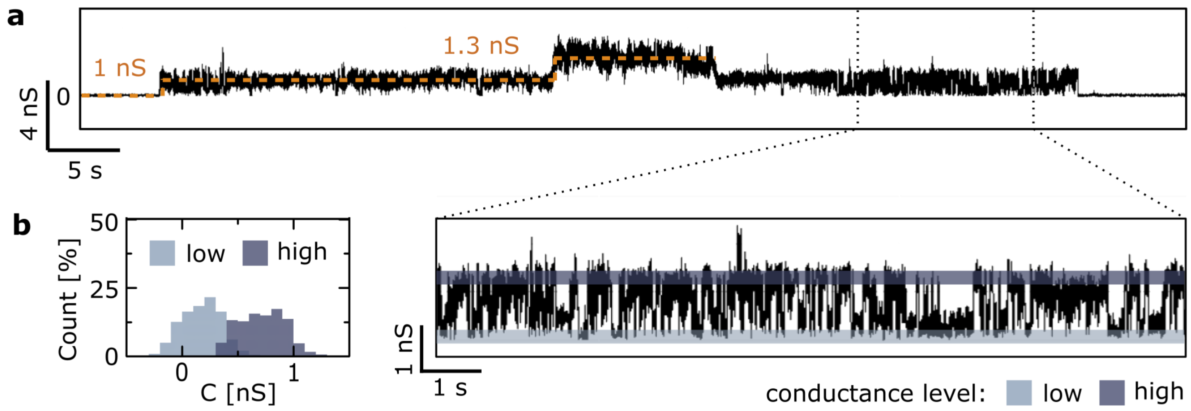


**Figure S9.** An example of two consecutive, well-defined insertions (a), followed by the nanostructures exiting the bilayer, illustrating the transient behavior of the pores. The further part of the traces is an example of the "gating" behavior (b) observed earlier for similar structures. During "gating", the pore oscillates between low and high ("closed" and "opened") conductance states. The concept of gating has been described in detail for natural ion channels, where similar single-molecule traces are observed. However, we note that the DNA nanostructure did not feature any specific gating mechanism, suggesting that the observed transient signal is initiated by unspecific structural changes in the membrane-spanning molecule.

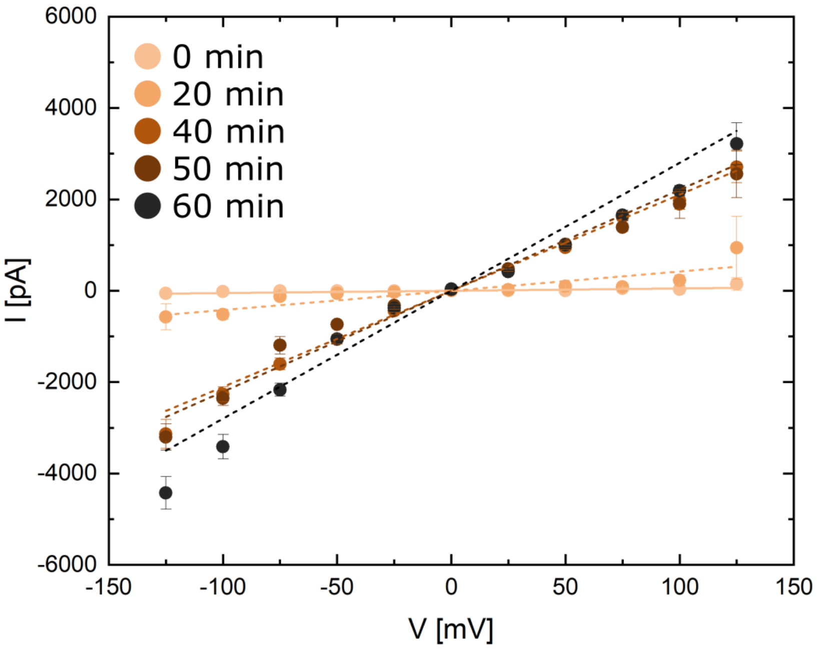


**Figure S10.** I-V curves collected for a shielded 2C structure during the measurement. The I-V curves were collected at given times during the measurements of current changes at 50 mV (analogous to the runs presented in Fig. 3), where "0 min" means before the start of the experiment. Error bars represent standard deviations from all the measured signals during the "sweep" (1.8 s). The dashed lines represent linear fit. While at higher voltages the data points diverge from the linear trend, at 50 mV (the voltage at which main experiments (Fig. 3) were performed), we assume ohmic behavior of the channels.

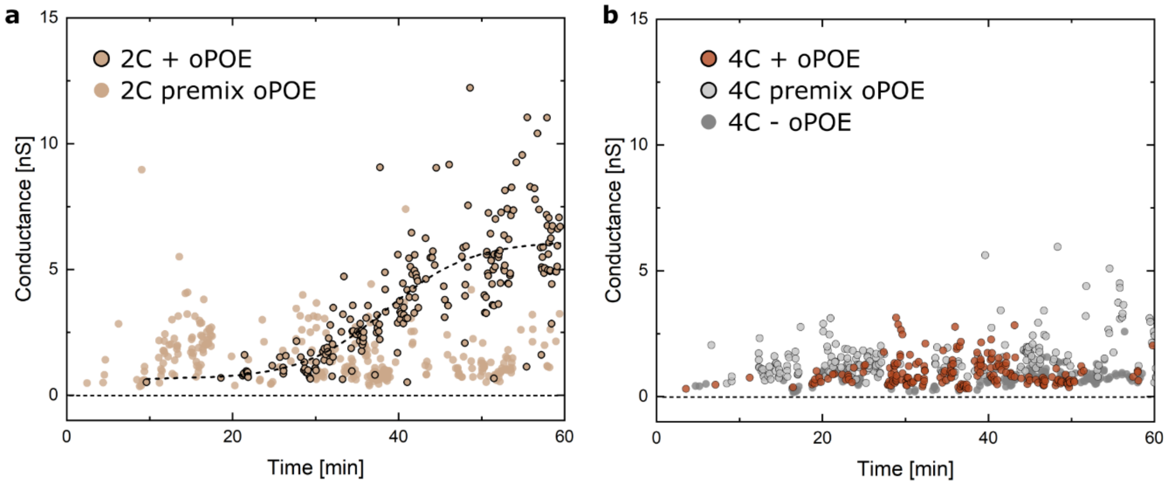


**Figure S11.** Scatter plots of discrete conductance changes (attributed to membrane insertions) vs. time from the beginning of the measurement, collected for shielded (a) 2C and (b) 4C samples. 2C without addition of oPOE did not produce any detectable signal. The data for "2C+oPOE" have been fitted with a sigmoidal curve to better illustrate the trend. The addition of oPOE, either premixed with the DNA or added separately, did not affect the insertions of 4C in a significant manner. However, we note that all samples premixed with oPOE produced signals earlier in the measurements than other samples.

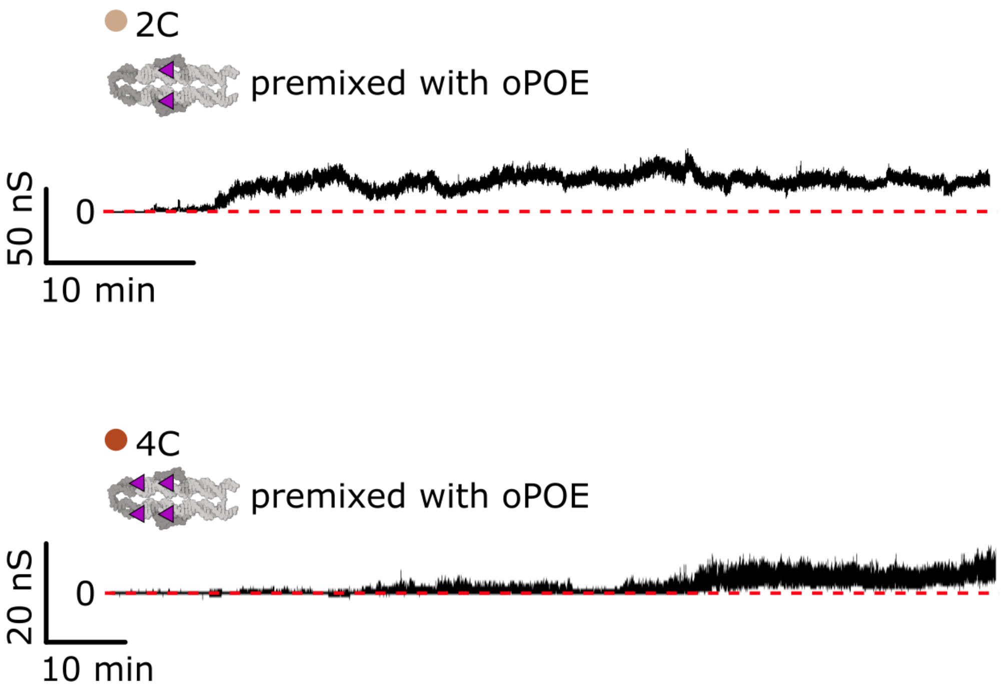


**Figure S12.** Conductance traces for 4H structures premixed with oPOE before the experiment. The final concentration of oPOE in the measurement chamber was the same as in other experiments (0.01%). Although 2C shows a higher insertion efficiency than aggregating 4C, the conductance values plateau and never reach the high level obtained for samples where oPOE was added to the chamber separately.

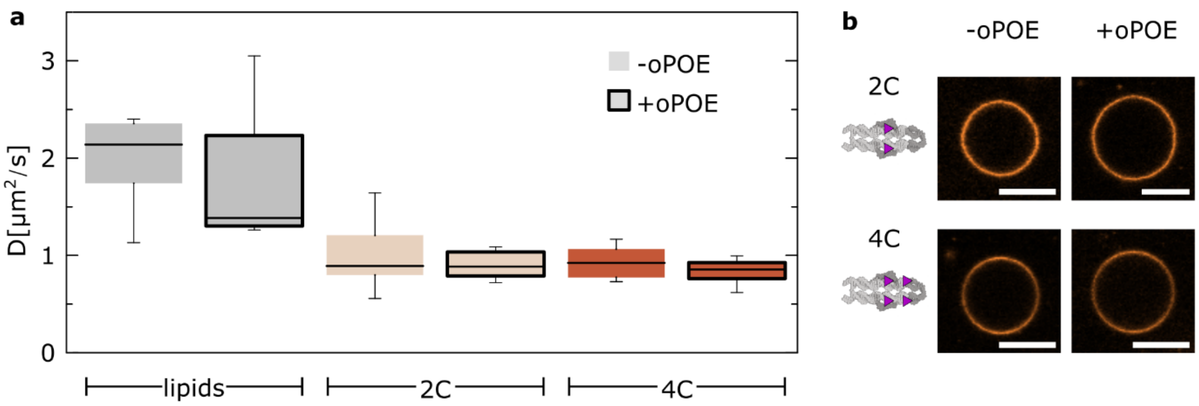


**Figure S13.** Confocal microscopy analysis of oPOE effects on a DNA–lipid system. (a) Box plots of diffusion coefficients collected for NBD-labelled PC lipids (no DNA addition) and Cy3-labelled DNA coating of the POPC vesicles, using fluorescence recovery after the photobleaching (FRAP) method, measured at room temperature. Values with (+oPOE) and without (-oPOE) the addition of the surfactant at a final concentration of 0.01% were recorded. Each measurement was repeated on at least 10 vesicles. (b) Representative micrographs of Cy3-labelled structures coating POPC vesicles in the absence and presence of 0.01% oPOE. Scale bars: 10 µm.

Table S1. Sequences of DNA strands forming 4H structures. An illustration of the design can be found in Fig. S12. “Modif.” stands for “Modification”. • represents the position of cholesterol. “Shielding” overhangs are highlighted in red.

| ***Strand*** | ***Sequence (5’ > 3’)*** | ***Length (nt)*** | ***Modif.*** |
|---|---|---|---|
| sc1 | TTTAGCCTAGTCAGCCGTTAAGTGCCA● | 27 | 3’ chol |
| sc2 | AGTCTGACCTGATTGAACGTAG● | 22 | 3’ chol |
| sc3 | CGTCTCCATAGCTACTTAGCTACAGATAGGTCCACAACCAGATTACACACTTT | 53 | |
| st1 | TTTTTTCAGACTTTTTTTTTTTTTTTTGGCACTTAACCTAT● | 41 | 3’ chol |
| st1 (no shield) | CAGACTTTTTTTTGGCACTTAACCTAT● | 27 | 3’ chol |
| st2 | TTTTTTGGAGACGTTTTTTTTTTTTTTCTACGTTCAATTTGTGGACCTATCTTT | 54 | |
| st2 (no shield) | GGAGACGTTTTTTCTACGTTCAATTTGTGGACCTATCTTT | 40 | |
| st3 | TTTTGTAGCTAAGTAGGGCTG | 21 | 5’ Cy3 |
| st4 | ACTAGGCTGTGTGTAATCTGGCAGG● | 25 | 3’ chol |
